# The Technologies, Applicability, and Trade-Offs of AI in Palliative Care for Older Adults: Scoping Review

**DOI:** 10.2196/100216

**Published:** 2026-08-31

**Authors:** Lei Huang, Menglu Xu, Ying Yang, Yi Tian, Zhongkang Xu, Yixuan Wang, Anning Hou, Lili Zhu, Yan Lin, Lina Wang, Yifei Zhao, Shuhong Wei, Peng Wang, Lin Peng

**Affiliations:** 1 School of Nursing, Henan Medical University, Xinxiang, China; 2 Outpatient Department, West China Hospital, Sichuan University, Chengdu, China; 3 Outpatient Department of Internal and Surgical Medicine, Union Hospital, Tongji Medical College, Huazhong University of Science and Technology, 1277 Jiefang Avenue, Wuhan, China, 1 19903735343

**Keywords:** AI, palliative care, older adults, applicability, scoping review

## Abstract

**Background:**

The rapid advancement of AI has introduced new opportunities for palliative care. However, its context-specific applicability and the trade-offs related to it use for older adults with multimorbidity, functional decline, and complex care needs remain unclear.

**Objective:**

This review aimed to characterize the applicability of AI in palliative care for older adults and synthesize its potential benefits and limitations.

**Methods:**

A scoping review was conducted following the framework of Arksey and O’Malley. Literature searches were performed in PubMed, Web of Science, CINAHL, Embase, and Scopus. Eligible studies were screened, and data were synthesized as a narrative synthesis incorporating thematic analysis.

**Results:**

Eleven studies were included, primarily comprising retrospective predictive model development and validation studies, as well as AI-based clinical information extraction studies. Applications were examined in hospital and community settings and drew on diverse routinely collected and population-based data sources, including electronic health records, clinical databases, administrative claims and health insurance databases, and population-based longitudinal survey datasets. Traditional machine learning, deep learning, and natural language processing approaches were applied. AI applications encompassed prediction and identification, monitoring and data integration, clinical decision support, and health care system optimization. Reported potential roles occurred across the data management, application performance, and clinical practice levels and included multisource information integration, more efficient data use, identification of potential palliative care beneficiaries and health risks, prognostic prediction, and quantitative support for clinical decision-making. Potential cost savings were suggested but not directly evaluated. Reported limitations relevant to real-world implementation included insufficient data reliability and availability, weak model generalizability, and restricted applicability. Human-centered limitations were infrequently examined and included difficulty recognizing patients’ emotions and the continuing need for human intervention.

**Conclusions:**

AI has the potential to support palliative care for older adults, but its clinical effectiveness and implementation effects remain uncertain. In this complex care context, the potential benefits of AI should be recognized while its inherent limitations are carefully considered. Future research should prioritize external model validation, real-world implementation studies, interoperable data systems, and the integration of patient-centered and contextual information. In clinical palliative care, AI should be positioned as an assistive tool, complementing rather than replacing clinical judgment and humanistic care.

## Introduction

### Background

The World Health Organization (WHO) has emphasized that palliative care should be integrated into national health systems to ensure comprehensive and continuous care for individuals with life-limiting illnesses [1]. Palliative care aims to improve quality of life through multidisciplinary management of physical symptoms, psychological distress, and spiritual needs while also supporting family caregivers throughout the end-of-life process [2,3]. As global population aging accelerates, the prevalence of chronic diseases and multimorbidity continues to increase, resulting in a growing demand for palliative care services among older adults [4,5]. However, service capacity has not kept pace with this increasing demand, creating substantial challenges for health care systems worldwide [6].

Older adults receiving palliative care often present with heterogeneous symptoms, fluctuating disease trajectories, and complex physical, psychological, and social needs, making assessment, prognostic evaluation, and individualized care planning particularly challenging [7,8]. These difficulties are compounded by shortages of specialized personnel, limited continuous monitoring, and insufficient psychosocial support, especially in home and community settings, where delayed recognition of clinical deterioration may compromise care quality and end-of-life experiences [9-12]. These challenges underscore the need for innovative approaches to improve the efficiency, precision, and accessibility of palliative care.

AI has emerged as a promising technology to address these challenges through large-scale data integration, predictive analytics, automated decision support, and continuous patient monitoring [13,14]. Early applications primarily focused on structured clinical data for risk prediction and survival estimation [15]. More recently, advances in machine learning, wearable devices, Internet of Things technologies, and generative AI have expanded AI applications to dynamic symptom monitoring, remote care, multimodal data analysis, workflow optimization, and personalized clinical decision support [16,17]. These developments have accelerated the digital transformation of palliative care and created new opportunities to improve care delivery for older adults.

### Research Questions and Objectives

Despite these advances, existing evidence remains fragmented. Most studies have focused on specific AI applications, such as mortality prediction, symptom deterioration alerts, or frailty identification, with considerable heterogeneity in algorithms, data sources, and implementation settings [18,19]. Furthermore, important issues, including model interpretability, ethical considerations, age-friendly design, humanistic care, and implementation challenges, have received limited and inconsistent attention [20,21]. Previous reviews have examined AI model applications, data sources, validation methods, generalizability, transparency, and reproducibility [22]. However, these reviews have not specifically focused on older adults, and the study populations were largely limited to patients with cancer. Furthermore, the identified AI applications have primarily focused on short-term mortality prediction, with limited exploration of AI’s broader applicability, advantages, and limitations. These gaps hinder a comprehensive and balanced understanding of AI in palliative care for older adults.

Given the rapid advancement of AI technologies and their growing relevance to health care, a comprehensive synthesis of current evidence is increasingly needed. These developments have broadened the potential role of AI beyond isolated prognostic models toward more continuous, data-informed, and potentially personalized support across the palliative care trajectory. However, whether these technologies can meaningfully address the complex and multidimensional needs of older adults while remaining clinically interpretable, ethically acceptable, equitable, and compatible with humanistic care remains insufficiently understood. Accordingly, this scoping review aims to systematically map AI applications in palliative care for older adults; synthesize their technical characteristics, applicability, advantages, and disadvantages; and identify gaps and priorities for future development. By integrating evidence across clinical, technological, and implementation dimensions, this review seeks to provide a more context-sensitive understanding of AI use in older adult palliative care and to inform clinical practice, health care decision-making, technology development, and future research.

## Methods

### Study Design

This study was conducted following the 5-stage scoping review framework proposed by Arksey and O’Malley [23], with methodological enhancements informed by Levac et al [24] and updated guidance from JBI. The review was reported in accordance with the PRISMA-ScR (Preferred Reporting Items for Systematic Reviews and Meta-Analyses extension for Scoping Reviews) guidelines [25]. A protocol was developed for this review, but it was not registered. A scoping review approach was selected because it is particularly suitable for mapping the scope, characteristics, and distribution of evidence in emerging and heterogeneous research fields. Given the diversity of study designs, AI technologies, and application contexts in palliative care, this methodology allowed for a comprehensive synthesis of existing evidence and the identification of key knowledge gaps.

### Search Strategy

A comprehensive literature search was conducted across 5 electronic databases, including PubMed, Web of Science, CINAHL, Embase, and Scopus. The search covered all records from database inception to July 15, 2025. The search strategy used free-text terms related to “palliative care,” “artificial intelligence,” and “older adults,” combined with the Boolean operators “AND” and “OR.” Search terms were adapted to the syntax and search fields of each database. To ensure transparency and reproducibility, the full search process, including database selection, search term development, and iterative refinement, was systematically documented. The detailed search strategies for each database are provided in Multimedia Appendix 1.

### Eligibility Criteria

Study selection was conducted based on predefined inclusion and exclusion criteria. Studies were included if they (1) focused on the applicability or trade-offs of AI in older adult palliative care; (2) adopted quantitative, qualitative, or mixed-methods designs; (3) were published in English or Chinese; and (4) involved older adults receiving care in palliative care units, nursing homes, hospitals, and home-based care settings. Studies were excluded if they (1) were commentaries, opinion papers, editorials, case reports, or book chapters without original data; (2) were not relevant to older adult palliative care; (3) did not provide substantive data or relevant findings; (4) had unavailable full texts; or (5) reported duplicate results already included in other studies.

### Data Extraction and Tabulation

Based on the included studies, a standardized data extraction form was developed and applied by the research team to systematically organize relevant information. The extracted variables included the first author and year of publication, country or region of the study, characteristics and sample size of participants, primary setting, algorithm, primary outcome, data sources, and performance measures. The data items were predefined based on the research questions and refined during the data extraction process when necessary. Before formal data extraction, the research team selected 3 included studies representing different clinical settings, data sources, and AI algorithms to pilot-test the data extraction form. Two reviewers independently extracted the relevant information and compared their entries item by item, with particular attention to potentially overlapping concepts. Based on issues identified during the pilot test, the research team revised the extraction form and clarified the data extraction criteria. Using the finalized form, 2 reviewers independently extracted data from all included studies. No additional assumptions or data transformations were applied, and extracted information was reported according to the characteristics of the original studies. All extracted data were cross-checked for accuracy and consistency before being entered into Microsoft Excel. The structured dataset was subsequently used to construct summary tables, providing a systematic foundation for the subsequent evidence synthesis.

### Data Synthesis and Reporting

A narrative synthesis incorporating thematic analysis was adopted [26]. Although all included studies were quantitative, substantial heterogeneity in algorithm types, data sources, performance measures, and application functions precluded statistical pooling or direct comparison of model performance. As this scoping review did not aim to estimate a common intervention effect, this approach was considered appropriate for the review purposes. First, each included study was assigned a numerical identifier according to its order of inclusion (eg, “01,” “02”). Extracted information was subsequently analyzed and compared across studies. Descriptive summaries were conducted to characterize the distribution and features of the included studies and to provide context for the subsequent synthesis. Guided by the predefined review questions, thematic analysis was used to organize and synthesize the extracted evidence. The 3 analytical domains of technologies, applicability, and trade-offs were established deductively, while the categories and subcategories within each domain were developed inductively from the included evidence. Theme development followed an iterative process. Two reviewers independently examined the extracted data and original studies and assigned initial codes to content relevant to the review questions. Codes with similar meanings or functions were then compared and grouped into preliminary categories and subcategories, which were repeatedly reviewed against the extracted data from all included studies to refine their boundaries and labels. A theme or category was retained only when it directly addressed a review question and had a clear conceptual boundary. When disagreements arose, the 2 reviewers re-examined the original studies, extracted data and relevant operational definitions, and resolved the disagreement through discussion. If consensus could not be reached, a third reviewer reviewed the source materials and made the final decision. Finally, synthesized findings were presented using narrative descriptions, tables, and figures to enhance transparency and facilitate interpretation. Consistent with methodological guidance for scoping reviews, no quality appraisal was conducted because this review aimed to map the characteristics and scope of the available evidence rather than assess methodological quality or the clinical effectiveness of interventions [25]. Accordingly, the findings were synthesized descriptively and interpreted cautiously in light of study heterogeneity and evidence limitations.

### Methodological Rigor

Consistency in data extraction and evidence synthesis was supported by a pilot-tested data extraction form, standardized operational definitions and extraction criteria, independent extraction and coding by 2 reviewers, and adjudication by a third reviewer when necessary. If the extraction criteria or coding rules were revised, all included studies were re-examined using the updated criteria. Given the substantial heterogeneity in clinical settings, data sources, and algorithm types, evidence was not grouped according to a single algorithm or model performance. Instead, within the 3 predefined analytical domains, evidence was categorized according to shared technical attributes, clinical settings and functions, and conceptual similarities. Model performance was reported descriptively without statistical pooling, ranking, or direct comparison. Findings recurring across different populations, settings, or study methods were summarized as cross-study commonalities.

## Results

### Study Selection

**Figure 1.**
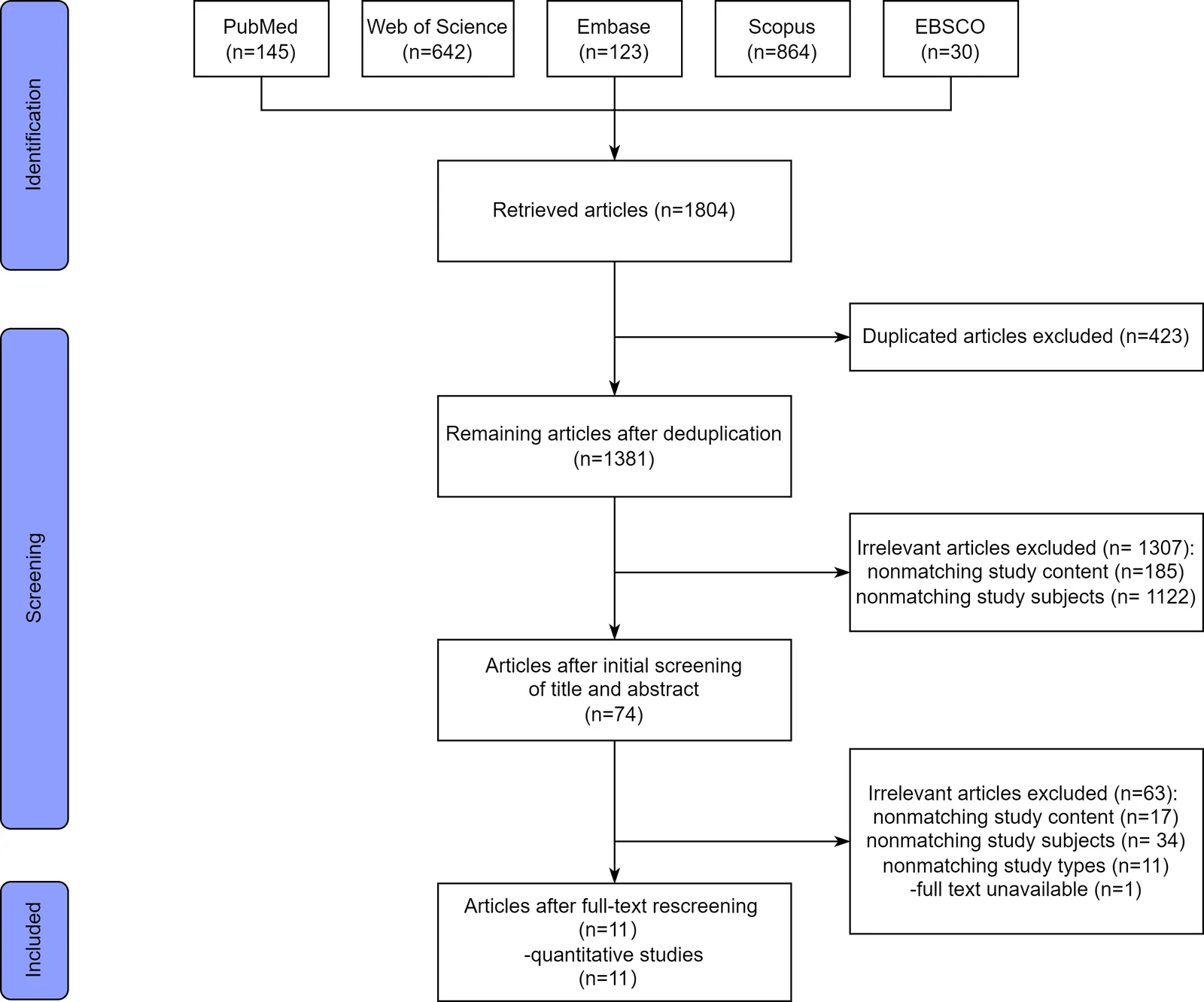
Preferred Reporting Items for Systematic Reviews and Meta-Analyses extension for Scoping Reviews flowchart.

A total of 1804 records were initially identified and imported into EndNote X9 (Clarivate Plc) for management. After removing duplicates, 1381 records remained. Title and abstract screening was conducted independently by 2 reviewers (LH and YY), resulting in the exclusion of 1307 studies that did not meet the inclusion criteria. The full texts of the remaining 74 studies were retrieved and independently assessed by the same 2 reviewers. Discrepancies at both screening stages were resolved through discussion to ensure consistency. Following detailed evaluation against the eligibility criteria, 11 studies were ultimately included in the final analysis. The screening process and detailed reasons for exclusion are presented in the PRISMA-ScR flow diagram (Figure 1).

### Study Characteristics

**Figure 2.**
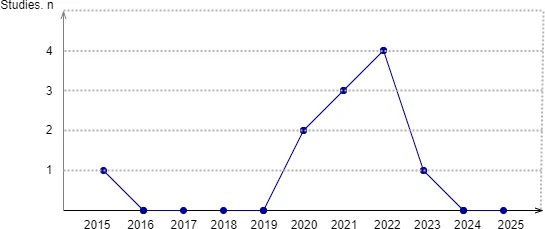
Number of included publications per year until 2025.

Eleven studies were included in this review, and their characteristics are summarized in Table 1. All included studies used quantitative designs, primarily involving retrospective predictive model development and validation, as well as AI-based clinical information extraction approaches. The study populations mainly consisted of older adults who were potentially at risk of requiring palliative or end-of-life care, including individuals with advanced cancer, dementia, severe illness, hip fractures, and other complex health conditions. Studies were conducted in both hospital and community settings. Sample sizes varied considerably, ranging from 117 participants in exploratory machine learning studies to more than 2.72 million records in a large-scale retrospective analysis. The included studies were published between 2015 and 2023, with the majority published between 2020 and 2023. The temporal distribution of the included studies is presented in Figure 2. Geographically, the studies were conducted in 3 countries, predominantly in the United States, followed by Spain and Sweden. The detailed geographical distribution of the included studies is presented in Table 2. The synthesized evidence was organized into the 3 predefined analytical domains of technologies, applicability, and trade-offs. Within these domains, categories and subcategories reflected shared technical attributes, clinical settings and functions, and reported advantages and disadvantages.

**Table 1.** Characteristics of the studies included in the revieww.

Author and year of publication	Country	Study population	Sample size	Primary setting	Algorithm	Primary outcome
Bowers et al [27], 2023	United States	Individuals aged ≥65 years in Medicare Advantage	318,774	Community	Light gradient boosting machine	1-year all-cause mortality
Lindvall et al [28], 2022	United States	Patients aged ≥65 years with advanced cancer	435	Oncology outpatient department and ward	Natural language processing	Advance care planning documentation
Qiao et al [29], 2022	United States	Patients with cancer aged ≥75 years	2,723,330	Emergency department	Gradient boosting machine; logistic regression; neural network	In-hospital mortality
Sullivan et al [30], 2022	United States	Patients with dementia aged ≥65 years	117	Community	Random forest	Hospice transition/use
Blanes-Selva et al [31], 2022	Spain	Hospitalized patients aged ≥65 years	19,753	Inpatient ward	Gradient boosting machine; random forest	Patients in need of palliative care; 1-year mortality
Cary et al [32], 2021	United States	Patients with hip fractures aged ≥65 years	17,140	Rehabilitation ward	Logistic regression; multilayer perceptron; neural network	30-day and 1-year all-cause mortality
Blanes-Selva et al [33], 2021	Spain	Hospitalized patients aged ≥65 years	19,753	Inpatient ward	Gradient boosting machine; deep neural network	1-year mortality; survival estimation; 1-year frailty
Macieira et al [34], 2021	United States	Inpatients aged ≥65 years	4354	Medical-surgical ward	Random forest	Classification of nursing care plan data into palliative care categories
Cao et al [35], 2020	Sweden	Patients aged ≥65 years undergoing emergency laparotomy	157	Emergency surgical ward	Logistic regression; random forest	90-day mortality
Udelsman et al [36], 2020	United States	Patients aged ≥75 years in the intensive care unit	1141	Intensive care unit	Deep neural networks	Documentation of care preferences
Makar et al [37], 2015	United States	Medicare beneficiaries with severe illnesses aged ≥65 years	80,000	Community	Random forest; naive Bayes; support vector machine; neural network; logistic regression; k-nearest neighbors	6-month mortality

**Table 2.** Clusterwise distribution of countries and number of studies.

Cluster name and countries	Studies, n
North America	8
United States	8
Europe	3
Spain	2
Sweden	1

### Technologies

#### Algorithm Type

The included studies used diverse AI approaches, which were mainly categorized into traditional machine learning, deep learning, and natural language processing (Table 3). Traditional machine learning was dominant and included the broadest range of algorithms, most commonly logistic regression, random forest, and gradient boosting variants [27,29-35,37]. Neural network–based models were used less frequently [29,32,33,36,37], while natural language processing was limited to 1 study examining unstructured clinical text [28]. Overall, the evidence centered on prediction and classification using structured data, with limited application to unstructured clinical information.

**Table 3.** Technical characteristics of AI in the included studies.

Category	Subcategory	Description	References
Algorithm type	Traditional machine learning	Logistic regression	[29,32,35,37]
		Random forest	[30,31,34,35,37]
		Naive Bayes	[37]
		Support vector machine	[37]
		Gradient boosting machine	[29,31,33]
		Light gradient boosting machine	[27]
		k-nearest neighbors	[37]
	Deep learning	Multilayer perceptron	[32]
		Neural networks	[29,32,33,36,37]
	Natural language processing	Natural language processing	[28]
Data sources	Electronic health records and clinical databases	Electronic health record databases from health care systems and hospitals	[27,28,33,34]
		MIMIC-III^a^ database	[36]
		University and Polytechnic La Fe Hospital electronic health record database	[31]
		Electronic medical records from a hospital	[35]
	Administrative and health insurance databases	Evernorth Health claims database	[27]
		Centers for Medicare and Medicaid Services administrative databases	[32]
		Medicare claims data	[27]
		Medicare administrative claims database	[37]
	Population-based surveys and public health datasets	National Emergency Department Sample	[29]
		National Health and Aging Trends Study	[30]
		National Study of Caregiving	[30]
Performance measures	Discrimination performance	Area under the curve	[27,29-33,35,37]
		Specificity	[30,32,33,35,36]
		Average precision	[27]
		Recall/sensitivity	[27,28,30,32-36]
	Classification performance	Accuracy	[28,30,32-36]
		Precision	[28,32,34,36]
		*F*_1_-score/*F* measure	[28,34,36]
	Clinical predictive value	Positive predictive value	[27,32]
		Negative predictive value	[27,32]
	Calibration performance	Calibration slope	[32]

a MIMIC: Medical Information Mart for Intensive Care.

#### Data Sources

The included studies used diverse data sources, which were mainly categorized into electronic health records and clinical databases, administrative claims and health insurance databases, and population-based surveys and public health datasets (Table 3). Most applications relied on routinely collected secondary data from electronic health records and clinical databases [27,28,31,33-36] or administrative claims and health insurance databases [27,32,37]. Population-based surveys and public health datasets were less frequently used [29,30]. Accordingly, the evidence base was largely derived from retrospective analyses of existing health data.

#### Performance Measures

The performance of AI models was evaluated using diverse metrics, which were mainly categorized into discrimination performance, classification performance, clinical predictive value, and calibration performance (Table 3). Model evaluation predominantly emphasized discrimination and classification performance [27-37]. Positive and negative predictive values were reported in 2 studies [27,32], whereas calibration was assessed in only 1 [32]. Thus, performance reporting focused primarily on discrimination and classification, with limited assessment of calibration and clinically oriented predictive values.

### Applicability of AI

#### Application Setting

The application of AI in palliative care for older adults was reported across diverse health care and care contexts, mainly including hospital and community environments (Table 4). Hospital applications were more varied, spanning oncology, emergency, inpatient, rehabilitation, medical-surgical, and intensive care settings [28,29,31-36], whereas community applications were reported in 3 studies [27,30,37].

**Table 4.** Applicability of AI in the included studies.

Category	Subcategory	Description	References
Care setting	Hospital	Oncology outpatient and ward	[28]
		Emergency department	[29,35]
		Inpatient ward	[31,33]
		Rehabilitation ward	[32]
		Medical-surgical ward	[34]
		Intensive care unit	[36]
	Community	Community	[27,30,37]
Functional roles	Predicting and identifying	Mortality risk prediction	[27,29-33,35,37]
		Survival estimation and frailty classification	[27,29-33,35,37]
		Risk stratification for individualized palliative care	[27,29,31,37]
	Monitoring and integrating	Real-time monitoring of vital signs and dynamic analysis of patient conditions	[28,37]
		Automated monitoring of adverse medical events	[28]
		Health monitoring for specific patient populations	[29,33,34,37]
		Automated classification and conversion of standardized nursing data	[34]
		Structuring, processing, and integrating multisource clinical data	[28,34,36]
	Clinical decision-making	Referrals for end-of-life care	[27,29,30,32,33]
		Investigating documented care preferences	[36]
		Explainable AI-supported model interpretation	[27,33]
		Bedside assessment of palliative care needs using smartphones or tablets	[31,33]
		Theoretical basis of accurate hierarchical triage in nursing institutions	[30]
	Optimizing medical systems	Optimized allocation of palliative care resources	[27,29-33]
		Evaluation and improvement of palliative care service outcomes	[27]
		Monitoring of clinical quality	[28]
		Identification of variations in physician care	[36]
		Providing the basis for optimizing resource allocation	[29,30]

#### Functional Roles

The functional roles of AI in palliative care for older adults were categorized into 4 domains: prediction and identification, monitoring and data integration, clinical decision support, and health care system optimization (Table 4). Prediction and identification constituted the dominant functional role, including mortality risk prediction, survival estimation, frailty classification, and risk stratification for individualized palliative care [27,29-33,35,37]. Monitoring and data integration functions involved real-time monitoring of patient conditions, adverse medical events, health status, and structuring and integrating clinical data from multiple sources [28,29,33,34,36,37]. Clinical decision support functions included supporting end-of-life care referrals, identifying documented care preferences, interpreting AI model outputs, and assessing palliative care needs [27,29-33,36]. At the health care system level, AI was applied to optimize palliative care resource allocation, evaluate service outcomes, monitor clinical quality, and identify variations in care delivery [27-33,36].

### Trade-Offs

#### Advantages

Reported advantages of AI applications in palliative care for older adults were identified at the data management, application performance, and clinical practice levels (Table 5). At the data management level, AI facilitated the integration of multisource information, improved the convenience and comprehensiveness of information collection, enabled faster access to relevant information, and simplified data use [27,28,34,37]. At the application performance level, AI supported beneficiary identification, earlier detection of deterioration and health risks, condition monitoring, and prediction of mortality, survival, frailty, and prognostic risk [27-33,35,37]. At the clinical practice level, AI was reported as potentially supporting individualized treatment adjustments, reducing subjectivity in clinical assessments, enhancing decision transparency, and providing quantitative evidence for care planning [27,29-33,35-37]. In addition, some studies suggested that AI-based approaches may contribute to health care cost savings through more efficient care planning and resource use [29,32]. Overall, the most direct evidence concerned information processing and prognostic assessment, whereas clinical and economic benefits were generally presented as potential rather than directly verified outcomes.

**Table 5.** Trade-offs of AI applications.

Category	Subcategory	Sub-subcategory	Description	References
Advantages of application	Data management level (information integration)	Information integration	Multisource information integration	[27,28,37]
			Convenient information collection	[27,28,37]
			More comprehensive information acquisition	[27,28,37]
			Faster access to information	[28]
			Simplifying data use	[34,37]
	Application performance level	Risk reduction	Identifies more potential beneficiaries	[27,29,32,37]
			Earlier detection of clinical deterioration and health risks	[27,28,30,31,33,35,37]
			More accurate monitoring of medical conditions	[29,30]
		Precise prediction	Prediction of mortality or survival time	[27,29,30,32,33,35,37]
			Prediction of the frailty index at 1 year	[31]
			Quantification of prognostic risk	[27,29,31-33,35,37]
	Clinical practice level	Clinical decision support	Tailored adjustments to treatment strategies	[29,30,32,37]
			Reducing subjectivity in clinical assessments	[36]
			Enhanced transparency in decision-making	[27,33]
			Provision of quantitative evidence to support personalized treatment	[27,29-31,33,35,37]
		Cost saving	Potential reduction in health care costs	[29,32]
Disadvantages of application	Data management level	Limited data reliability	Data gaps	[27,29,33,35]
			Data bias	[29,30,33]
			Data has time lag	[32]
			Insufficient data standardization	[33]
			Significant differences in electronic health record formats across health care institutions	[28]
			Lack of external validation dataset	[35,36]
		Insufficient data	Limited data sources	[30,31]
			Data omits lab, medication and sociobehavioral variables	[32]
			Restricted to written documentation, missing unrecorded verbal discussions	[36]
	Application performance level	Weak extrapolation	Limited scope of model	[27,35,37]
			Only associations identified without causal inference	[36]
			Lack of interpretability	[29,33]
		Restricted performance	Low model sensitivity	[35]
			Difficulty in maintenance and updating	[31]
	Clinical practice level	Lack of humanity	Inability to accurately identify patient emotions	[34]
		Limited independent decision-making	Clinical integration requires manual intervention	[33]

#### Disadvantages

Reported limitations also occurred across the data management, application performance, and clinical practice levels (Table 5). At the data management level, limitations included data gaps, bias, time lag, inadequate standardization, heterogeneous electronic health record formats, limited data sources, and missing important clinical or contextual information [27-33,35,36]. At the application performance level, limitations included weak model extrapolation, restricted model scope, insufficient interpretability, limited sensitivity, difficulties in model maintenance and updating, and the inability to establish causal relationships based solely on associations [27,29,31,33,35-37]. At the clinical practice level, less frequently reported human-centered limitations concerned difficulty recognizing patient emotions and the continuing need for human intervention during clinical integration [33,34]. Overall, the reported limitations extended from data quality and model performance to clinical integration, while human-centered constraints received comparatively limited attention.

## Discussion

### Principal Findings

This scoping review synthesized 11 studies published between 2015 and 2023 to examine the applicability and trade-offs of AI in palliative care for older adults. All included studies adopted quantitative designs, with retrospective predictive model development and validation studies and AI-based clinical information extraction being the predominant approaches. AI applications were investigated across both community and hospital-based care settings, with applications primarily focused on 4 domains: risk prediction and identification, patient monitoring and data integration, clinical decision support, and health care system optimization. Traditional machine learning methods remained the dominant approaches, supplemented by deep learning and natural language processing techniques. The data sources used for model development mainly included electronic health records, administrative claims databases, and population-based cohort datasets. The trade-off analysis indicated that the included studies reported potential roles for AI in information integration, prognostic prediction, risk identification, clinical decision support, and more efficient resource use; however, these roles were not directly verified as improvements in clinical outcomes, workflow, or health care costs. AI application remains constrained by insufficient data reliability and availability, limited model generalizability and scope of application, and insufficient interpretability. Human-centered limitations, including difficulty recognizing patient emotions and the continuing need for human intervention, were reported in only a small number of studies.

### Comparison With Prior Work

Current evidence has primarily demonstrated the technical feasibility and computational performance of AI models, while real-world implementation, patient outcomes, workflow changes, cost-effectiveness, and patient-level benefits have rarely been evaluated. Therefore, the reported advantages of AI, including earlier identification of high-risk individuals, individualized decision support, and optimization of health care resource allocation, should currently be interpreted as potential benefits rather than established clinical effects. Limitations related to data quality, model generalizability, and algorithm interpretability have been frequently reported. By contrast, the ability of AI to account for patients’ psychosocial conditions, emotions, and value preferences has received limited investigation. Overall, current evidence suggests that AI should be regarded as an adjunctive tool within clinician-led, human-centered palliative care rather than a substitute for the core elements of such care.

This review indicates that AI applications in older adult palliative care remain an emerging research area that has attracted increasing attention in recent years. Unlike previous disease-specific studies that predominantly focused on patients with advanced cancer [38,39], the included studies extended AI applications to older adults with diverse palliative care needs, including dementia, hip fractures, multimorbidity, severe illnesses, and emergency surgical conditions. This broader population coverage indicates increasing recognition of the heterogeneous health conditions associated with palliative care needs in older adults. Furthermore, AI research has been conducted across diverse health care environments and has used various data sources, including electronic health records, clinical databases, administrative claims databases, and population-based longitudinal survey datasets.

Most included studies were published between 2020 and 2023, suggesting recent research interest, although the small evidence base does not establish a sustained growth trend. Across the evidence base, traditional machine learning was supplemented by a smaller number of studies using deep learning and natural language processing to analyze more complex clinical information [40,41]. However, despite the expanding application scope and diversification of AI approaches, the maturity of evidence and level of clinical integration remain limited [42]. From a geographic perspective, AI research in older adult palliative care remains concentrated in a small number of high-income countries. The studies included in this review were conducted only in the United States, Spain, and Sweden, with the United States accounting for the majority of studies. No studies from low- or middle-income countries met the inclusion criteria. Previous reviews have similarly reported that AI research in palliative care is concentrated in North America and Europe. This distribution may reflect differences in health care data infrastructure and research capacity [43]. In addition, unlike broader scoping reviews of clinical AI applications that have reported higher-level evidence, including randomized controlled trials, for evaluating AI interventions [44], AI research in older adult palliative care remains largely exploratory. Current studies mainly focus on retrospective predictive modeling and proof-of-concept analyses. The absence of randomized controlled trials, implementation studies, and real-world effectiveness evaluations in this review suggests that AI applications remain primarily at the stages of model development and performance assessment, leaving their clinical usefulness and implementation effects uncertain.

This review demonstrated that AI applications in older adult palliative care have been investigated across diverse settings, including hospitals and communities, indicating their potential relevance to more than one care context. However, evidence from different settings does not establish that AI improves transitions between settings or continuity of care. In addition, this review identified a range of AI approaches, including traditional machine learning, deep learning, and natural language processing. Traditional machine learning methods remained predominant, consistent with findings from 2 previous studies [45,46]. This may reflect that current AI applications remain largely focused on structured data analysis and predictive tasks. Regarding evaluation metrics, consistent with the findings of Li et al [45], the included studies primarily emphasized discrimination and classification performance, reflecting that current research remains focused on model accuracy and technical validation rather than evaluation of clinical effectiveness.

A key finding of this review is the systematic characterization of the functional roles of AI in older adult palliative care. Currently, AI in this field primarily serves as a tool for assisted identification and decision support rather than direct involvement in care delivery. Existing studies have mainly focused on prediction and identification tasks, including mortality prediction, survival estimation, frailty classification, and assessment of palliative care needs. The potential value of these applications lies in supporting the identification of patients who may benefit from palliative care and providing additional information for resource planning and clinical decision-making. However, this risk-oriented application pattern shows that the existing evidence primarily emphasizes identifying who may require palliative care, whereas the use of AI to support care aligned with patients’ values, preferences, and individual needs remains underexplored. Previous research in geriatric care and nursing has described potential roles for AI beyond prediction, including health monitoring, support for continuity of care, resource coordination, workflow assistance, and professional development [47,48]. In contrast, this review found that although AI applications in older adult palliative care have begun to incorporate patient monitoring, multidimensional clinical information integration, end-of-life decision support, and health care resource optimization, these functions were less frequently examined, and their effects on care processes and outcomes were not directly established. Therefore, future AI applications should further integrate patients’ subjective experiences, care goals, and contextual information and examine whether these capabilities can extend AI beyond risk identification to support individualized care planning, clinician-patient communication, and patient-centered palliative care across different settings [49,50].

Another key finding is that the reported potential and limitations of AI in palliative care for older adults coexist. On the one hand, AI may support patient identification and provide additional information for care-related decisions. By integrating multidimensional clinical information, AI models may help identify patients who may benefit from palliative care, predict disease trajectories and adverse outcomes, and provide information that could inform individualized care planning. This potential role may be relevant for older adults, whose health status is often shaped by multimorbidity, functional decline, and complex social factors, making it difficult for traditional assessment approaches to fully capture their dynamic needs [51,52]. Some included studies proposed that information integration and resource optimization could reduce clinicians’ data-processing burden, improve workflow efficiency, or lower health care costs. However, these outcomes were not directly evaluated, and the included evidence did not demonstrate improvements in continuity of care.

On the other hand, these potential roles do not imply that AI can replace clinical judgment. The included studies reported limitations involving insufficient data quality, limited model generalizability, inadequate interpretability, and challenges in clinical integration. More importantly, palliative care is not merely a process of risk prediction but a comprehensive care practice involving patients’ values, emotional needs, family relationships, and life meanings, dimensions of human experience that remain difficult for current algorithms to adequately understand [53,54]. These dimensions were rarely incorporated into or evaluated by the AI applications included in this review. Therefore, future AI development should extend beyond improving model performance to strengthen data quality and promote human-AI collaboration that integrates algorithmic capabilities with clinical judgment and humanistic care. Prospective, multicenter external validation and real-world implementation studies across diverse health care systems should directly evaluate clinical outcomes, patient and family experiences, workflow, continuity of care, and economic outcomes. AI should inform, rather than replace, palliative care decision-making.

### Limitations

This study has several limitations. First, all included studies were quantitative and primarily model-oriented, primarily focusing on the development and validation of AI models for prediction or classification, whereas longitudinal evaluations of implementation and clinical outcomes, intervention studies, and qualitative investigations of patient and family experiences were scarce. This restricted the available evidence on real-world integration, sustained use, clinical impact, and human-centered experiences of AI in palliative care. Second, although a comprehensive search was conducted across 5 major databases, only English-language studies were included, and the eligible studies were concentrated in Europe and North America; no studies from other continents met the inclusion criteria. This geographical concentration may limit the transferability of the findings across different health care systems and sociocultural contexts. Finally, consistent with the purpose of a scoping review, this study mapped the scope and characteristics of the available evidence but did not assess its methodological quality or certainty. Accordingly, the findings should not be interpreted as conclusions about the strength of evidence or comparative effectiveness of specific AI applications. Future research should include older adults with more diverse clinical, socioeconomic, and sociocultural backgrounds, particularly those from underrepresented regions, and employ longitudinal and interventional designs alongside implementation studies.

### Conclusion

This scoping review synthesized evidence from 11 studies and characterized the technical features, context-specific applicability, and trade-offs of AI applications in palliative care for older adults. Current research remains exploratory and is largely focused on retrospective predictive modeling, model validation, and clinical information extraction; evidence regarding real-world implementation, clinical effectiveness, and sustained use remains limited. AI applications have been explored in both community and hospital settings, encompassing prediction and identification, monitoring and data integration, clinical decision support, and health care system optimization. Across the data management, application performance, and clinical practice levels, the included studies reported potential roles for AI in multisource information integration, data use, risk identification, prognostic estimation, and decision support. However, AI’s effects on patient outcomes, care processes, continuity of care, clinician workload, and health care costs were not directly established.

Reported limitations that may hinder real-world translation included insufficient data reliability and availability, weak model generalizability, restricted applicability, and inadequate interpretability. Human-centered aspects received limited attention, with only a small number of studies reporting difficulty recognizing patients’ emotions and the continuing need for human intervention. Therefore, in complex palliative care contexts, AI should be positioned as a supportive tool that informs clinical decision-making and complements, rather than replaces, professional judgment and humanistic care. Future research should prioritize multicenter, longitudinal, real-world, and implementation-oriented studies, with particular attention to external validation, data interoperability, and the integration of patient-centered information.

## Supplementary material

10.2196/100216 Multimedia Appendix 1 Search strategies for each database.

10.2196/100216 Checklist 1 PRISMA-ScR checklist.

## Abbreviations

PRISMA-ScR: Preferred Reporting Items for Systematic Reviews and Meta-Analyses extension for Scoping Reviews
WHO: World Health Organization
