# Supplementary material for: The Technologies, Applicability, and Trade-Offs of AI in Palliative Care for Older Adults: Scoping Review
Source: J Med Internet Res. 2026 Aug 31;28:e100216. doi: 10.2196/100216 (PMC13528606; doi:10.2196/100216)
Supplement: Supplementary file 1 [file jmir-v28-e100216-s001.docx]

Appendix I Search strategies for each database

| Database | Search Terms |
| --- | --- |
| Web of science (n=642) | #1 TS=(AI OR "machine learning" OR "model interpretation" OR "model visualization" OR "deep learning")  #2 TS=(palliative OR terminal OR end-of-life OR hospice)  #3 TS=(ageing OR aging OR aged OR old* OR elder* OR senior)  #4 #1 AND #2 AND #3  Limit to (Article/Review/Early Access and English/Chinese language) |
| Pubmed (n=145) | #1 "ageing"[Title/Abstract] OR "aging"[Title/Abstract] OR "aged"[Title/Abstract] OR "old"[Title/Abstract] OR "older"[Title/Abstract] OR "oldest"[Title/Abstract] OR "elder*"[Title/Abstract] OR "senior"[Title/Abstract]  #2 "AI"[Title/Abstract] OR "machine learning"[Title/Abstract] OR "model interpretation"[Title/Abstract] OR "model visualization"[Title/Abstract] OR "deep learning"[Title/Abstract]  #3 "palliative"[Title/Abstract] OR "terminal"[Title/Abstract] OR "end-of-life"[Title/Abstract] OR "hospice"[Title/Abstract]  #4 #1 AND #2 AND #3  Filters:English/Chinese language |
| Ovid-Embase (n=123) | #1 ('ai':ti,ab OR 'machine learning':ti,ab OR 'model interpretation':ti,ab OR 'model visualization':ti,ab OR 'deep learning':ti,ab)  #2 ('palliative':ti,ab OR 'terminal':ti,ab OR 'end-of-life':ti,ab OR 'hospice':ti,ab)  #3 ('ageing':ti,ab OR 'aging':ti,ab OR 'aged':ti,ab OR 'old*':ti,ab OR 'elder*':ti,ab OR 'senior':ti,ab)  #4 ('article'/it OR 'article in press'/it OR 'data papers'/it OR 'review'/it OR 'short survey'/it OR 'preprint'/it)  #5 (chinese:la OR english:la)  #6 [abstracts]/lim  #7 #1 AND #2 AND #3 AND #4 AND #5 AND #6 |
| Scopus (n=864) | #1 TITLE-ABS-KEY(AI OR "machine learning" OR "model interpretation" OR "model visualization" OR "deep learning")  #2 TITLE-ABS-KEY(palliative OR terminal OR end-of-life OR hospice)  #3 TITLE-ABS-KEY(ageing OR aging OR aged OR old* OR elder* OR senior)  #4 #1 AND #2 AND #3  Limit to (Article/Review/Short survey and English/Chinese language) |
| EBSCO-CINAHL (n=30) | #1 XB("AI" OR "machine learning" OR "model interpretation" OR "model visualization" OR "deep learning")  #2 XB("palliative" OR "terminal" OR "end-of-life" OR "hospice")  #3 XB("ageing" OR "aging" OR "aged" OR "old*" OR "elder*" OR "senior")  #4 #1 AND #2 AND #3  Limiters: English or Chinese language |
